# Balanced Mix Design and Performance Analysis of High-Modulus Asphalt Mixtures

**DOI:** 10.3390/ma19132777

**Published:** 2026-06-30

**Authors:** Qirong Li, Jiwei Liu, Jilong Yang, Xinquan Xu, Xinhai Liu, Peiwen Hao, Ningbo Li

**Affiliations:** 1 Guangdong Road and Bridge Construction and Development Co., Ltd., Guangzhou 510623, China; liqirong1985@gmail.com (Q.L.); lnbwudi@163.com (J.L.); 2 School of Highway, Chang’an University, Xi’an 710064, China; 3 Guangdong Jiaoke Technology Development Co., Ltd., Guangzhou 510550, China; xuxinquan998@126.com (X.X.); liuxinhai8721@sina.cn (X.L.); 4 School of Highway Engineering, Shaanxi College of Communication Technology, Xi’an 710018, China; 2020021049@chd.edu.cn

**Keywords:** asphalt mixture, balanced mix design, pavement performance, balanced evaluation, fatigue performance

## Abstract

The objective of this study was to design mixtures that satisfy multiple performance criteria to build long-life pavement. A balanced mix design was employed to optimize AC-16, BBME-13, and EME-20 mixtures. The initial asphalt contents were determined using 4% air voids for the AC-16 mixture and the abundance coefficient *K* for BBME-13 and EME-20. Mixtures with two additional asphalt contents were also tested. A uniaxial penetration test, semi-circle bending test (to measure the flexibility index), and semi-circle bending test (to measure fracture energy) were used as performance tests. Then, a performance space diagram was created for a balanced analysis of the mixtures’ high-, intermediate-, and low-temperature performance. Finally, fatigue performance was verified. The results show that BMD could be used to evaluate different asphalt mixtures. The 20# BBME-13 (5.1%, 5.4%, and 5.7%), 50# BBME-13 5.4%, and 20# AC-16 4.7% mixtures had well-balanced performances and are recommended. The BBME-13 and EME-20 mixtures had higher fatigue lives and lower sensitivity to stress than the AC-16 mixture. For the BBME-13 and AC-16 mixtures, reducing the asphalt grade significantly increased fatigue life and decreased the sensitivity to stress. At a low stress ratio, the 20# EME-20 mixture had the best fatigue performance, whereas at a high stress ratio, the 20# BBME-13 mixture had the best fatigue performance.

## 1. Introduction

High-modulus asphalt concrete (HMAC) is being increasingly applied in asphalt pavement construction worldwide due to its excellent resistance to rutting and fatigue cracking, which is essential for producing long-life pavements that are subjected to heavy traffic loads [1,2,3,4,5]. HMAC is defined as an asphalt mixture with a high modulus or stiffness at intermediate temperatures (14,000 MPa at 15 °C and a 10 Hz loading frequency). Furthermore, it can be classified into three types based on the preparation method: mixtures produced using low-grade hard asphalt, self-blended asphalt, and polyolefin-modified mixtures [6]. In practice, HMAC is often designed as a wearing course that requires high abrasion resistance or as a base course that requires high rutting resistance in pavement structures. The design of high-modulus asphalt mixtures is widely based on the Marshall mix design where the proportions are determined according to volumetric parameters, flow values, and Marshall stability requirements. Although performance tests are conducted at the optimum asphalt content, the optimal air void content (AV) does not necessarily ensure optimal performance. Furthermore, the voids in mineral aggregate (VMAs) are highly dependent on the aggregate bulk specific gravity (Gsb). The measurement of Gsb is inaccurate and variable because of poor reproducibility [7]. In addition, standard volumetric parameter values might not apply to all mixes. In some pavements subjected to serious rutting and thermal cracking distresses, a balance between rutting and cracking requirements is required [8].

To address these limitations, some highway agencies have begun to adopt balanced mix design (BMD) in the mix design process as the results are believed to correlate better with field performance [9]. BMD is defined as an “asphalt mix design using performance tests on appropriately conditioned specimens that address multiple modes of distress taking into consideration mix aging, traffic, climate and location within the pavement structure”, which was proposed by the federal highway administration (FHWA) expert task group on mixes and construction. Moreover, AASHTO PP 105-20 [10] offers a detailed standard, which includes four approaches. Virginia [11] used the Cantabro test to measure durability and asphalt pavement alliance (APA) to evaluate rutting performance. These studies indicate that BMD improves the correlation between laboratory design and field performance.

Researchers have employed the BMD method, focusing primarily on the high-temperature rutting performance of asphalt mixtures as well as the monotonic and cyclic fatigue crack propagation performance at intermediate temperatures. Nebraska [12] used the semi-circle bending (SCB) test to assess thermal cracking performance and used G-stability to assess the rutting performance. Cooper et al. used the SCB test to evaluate the intermediate-temperature cracking resistance of asphalt mixtures, and their study indicated that the BMD approaches can produce superior asphalt mixtures [13]. Texas [14] has developed a specialized balanced design program to measure the laboratory performance of the Hamburg wheel-tracking test (HWTT) and overlay testers (OTs). However, research on the BMD method for high-modulus asphalt mixtures remains relatively scarce, particularly regarding the conversion of French BBME (wearing course) and EME (base course) gradations to Chinese specifications. Second, although the HWTT is an effective method for evaluating the coupled high-temperature stability and moisture damage resistance of asphalt mixtures, it has disadvantages such as time-consuming procedures, expensive equipment, and sensitivity to temperature and loading rate. Moreover, its load level (705 ± 4.5 N) deviates from actual overloaded traffic conditions, leading to low reliability of the evaluation results [15]. Meanwhile, BMD studies that comprehensively consider high-, intermediate-, and low-temperature performance remain insufficient. To address the above issues, a high-temperature performance evaluation method—the uniaxial penetration strength test—which is simple to prepare, fast to perform, and highly reliable, has been widely applied in China [16,17].

Moreover, performance space diagrams (PSDs), as a visualization tool for BMD, has been applied to balance the performance of asphalt mixtures containing RAP from different sources and contents [18,19,20]. PSDs simultaneously present multiple key performance indicators in a two- or three-dimensional coordinate system, enabling intuitive evaluation and optimization of mixture composition for balanced mix design.

This study utilized BMDs to optimize AC-16, BBME-13, and EME-20 high-modulus asphalt mixtures. The initial asphalt contents were selected based on volumetric parameters. Then, the results from performance tests, the semi-circular bending test, and the SCB fracture test were used to create a performance space diagram for a balanced analysis. Finally, the fatigue performance of the mixtures was verified. This approach provides a more reliable method for optimizing asphalt mixtures, particularly those with a high modulus, and contributes to the development of BMDs for high-modulus asphalt mixtures.

## 2. Materials and Methods

### 2.1. Materials

According to the relevant requirements of specification JTG 3410-2025 [21], the 20# and 50# hard asphalts (20 and 50 penetration grades, respectively) produced by Petro China Karamay Petrochemical Co., Ltd. (Xinjiang, China) were selected. Their technical properties are listed in Table 1.

According to the relevant requirements of JTG 3432-2024 [22], basalt was selected as the coarse and fine aggregates, and the filler was mineral powder processed from limestone. The aggregate properties are listed in Table 2.

### 2.2. Balanced Mix Designs for Asphalt Mixtures

Based on the balanced design method, this study designed high-modulus asphalt mixtures using the AC-16 gradation from current Chinese specifications (JTG F40-2004) [23], as well as the BBME-13 and EME-20 gradations from French specifications [24].

#### 2.2.1. AC-16 Balanced Mix Design

For the 20# AC-16 and 50# AC-16 mixtures, the initial asphalt content was based on a 4% AV content. Then, the asphalt contents were changed by ±0.5% and pavement performance tests were conducted. The composition of the AC-16 asphalt mixture is given in Table 3.

**Figure 1:**
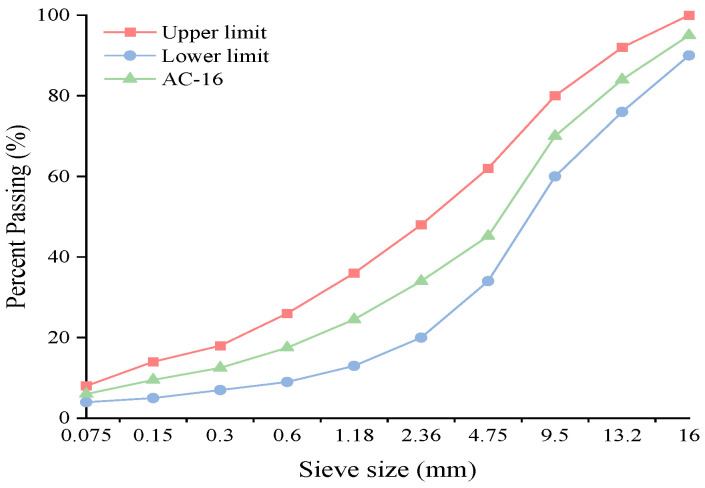
Gradation curve of AC-16.

The designed AC-16 gradation is shown in Figure 1.

The Superpave gyration compactor (SGC) method was used to mold a specimen with a 150 mm diameter. The drilled cylindrical specimens had a 100 mm diameter and 100 mm height. The volumetric parameters are listed in Table 4 and Table 5.

#### 2.2.2. BBME-13 and EME-20 Balanced Mix Designs

The BBME-13 and EME-20 gradations were designed to satisfy the Chinese specifications and were based on the French standard. The minimum asphalt content was determined using the abundance coefficient, and two additional asphalt contents around this value were also analyzed.

Due to the differences in sieve sizes between the French and Chinese standards, an interpolation method was employed to convert the passing percentages from the French system to the corresponding Chinese sieve sizes. The resulting gradations for BBME-13 and EME-20 are presented in Table 6.

**Figure 2:**
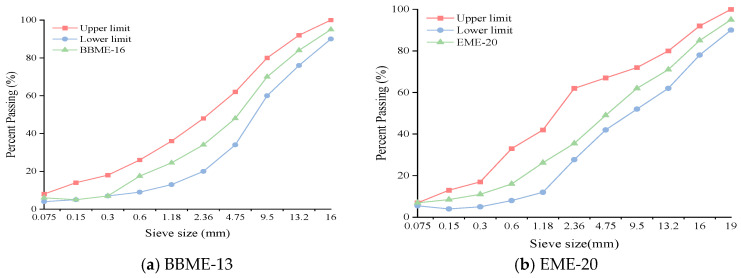
Gradation curve of asphalt mixtures.

The BBME-13 and EME-20 gradation results are shown in Figure 2.

In the design of France high-modulus asphalt mixtures, the minimum asphalt content is determined based on the abundance coefficient *K*, which is calculated using Formulas (1) and (2):

(1) $$K=\frac{TL}{a\sqrt[{5}]{\sum }}$$

(2) 100∑=0.25G+2.3S+12s+135f

where

*TL*—bitumen–aggregate ratio;

$\alpha =\frac{2.65}{G_{se}}\left(\;G_{se}\;\mathrm{is}\;\mathrm{the}\;\mathrm{effective}\;\mathrm{relative}\;\mathrm{density}\;\mathrm{of}\;\mathrm{aggregate}\right)$;

*G*—proportion of aggregate larger than 6.3 mm (%);

*S*—proportion of aggregate between 0.25 and 6.3 mm (%);

*s*—proportion of aggregate between 0.063 and 0.25 mm (%);

*f*—proportion of aggregate smaller than 0.063 mm (%).

The calculated *K* parameters are listed in Table 7.

The *K* of BBME-13 and EME-20 should not be less than 3.3 and 3.4, respectively, based on the France standard. The calculated minimum asphalt contents of BBME-13 and EME-20 are 5.01% and 5.3%. Combined with engineering experience, 5.1%, 5.4% and 5.7% contents were selected for BBME-13, while 5.3%, 5.8%, and 6.3% were selected for EME-20. The volumetric parameters are listed in Table 8.

### 2.3. Experiments

BMD selects the design with the shortest operation time and higher accuracy in performance tests. The SGC method is believed to correlate better with field performance and was applied in this study. Based on traffic levels, 75 gyrations were selected for testing AC-16. Based on the “LPC Bituminous Mixtures Design Guide”, 80 and 120 gyrations were selected to test BBME-13 and EME-20, respectively. The selected performance tests are shown in Table 9. Specimen preparation and testing were carried out in accordance with JTG 3410-2025. The performance tests were conducted using a UTM-100 testing machine (Industrial Physics, Shanghai, China), with relevant parameters configured through the UTS-024 module. The mixtures of the same gradation but different asphalt types were prepared under the same conditions.

Because performance test standards have not been established, methods for distinguishing between different asphalt mixtures were applied. For the uniaxial penetration test, if the performance of an asphalt mixture was better than the control mixture, the asphalt mixture was considered to satisfy the standard. Based on the literature [25], the minimum uniaxial penetration strength of common asphalt mixtures (70# AC-13, AC-16, and AC-20) is 0.6 MPa. Therefore, 0.6 MPa was chosen as the criterion. A uniaxial penetration strength below 0.6 MPa was defined as poor; a value between 0.6 and 1.0 MPa was defined as good; and a value above 1.0 MPa was defined as excellent.

To evaluate the intermediate-temperature (25 °C) cracking resistance of the asphalt mixtures, the flexibility index (FI) was determined. According to Ref. [26], values of 0–12, 12–40, and above 40 are classified as poor, good, and excellent, respectively. Moreover, to evaluate the fracture energy performance of the different asphalt mixtures, the following classification criteria were established according to the data distribution in previous studies [27,28]: a value between 0 and 2000 J/m^2^ was defined as poor; a value between 2000 and 4000 J/m^2^ was defined as good; and a value above 4000 J/m^2^ was defined as excellent.

Regarding low-temperature (−10 °C) anti-cracking performance, a value between 0 and 1 J was defined as poor; a value between 1 and 2 J was defined as good; and a value above 2 J was defined as excellent.

The fatigue performance of the high-modulus asphalt mixtures was evaluated using the SCB fatigue test. Based on the literature, four stress ratios (0.2, 0.3, 0.4, and 0.5) were selected for the SCB fatigue test [29,30]. Under the stress-controlled mode, the fatigue life of the HMACs was defined as the number of cycles at which the stiffness modulus decreases to 50% of its initial value (Equation (3)):

(3) $$N_{f}=K{\left(\frac{1}{\sigma_{0}}\right)}^{n}$$

where $N_{f}$ is the number of load applications at failure, $\sigma_{0}$ is the initial flexural tensile stress (MPa), and K and *n* are fitting parameters.

## 3. Results and Discussion

### 3.1. Performance Space Diagram Analysis for Multiple Distresses

**Figure 3:**
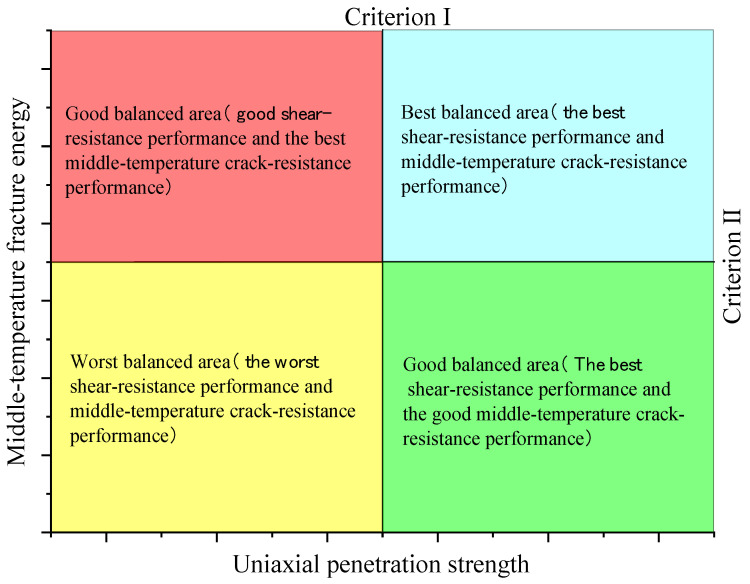
Performance space diagram.

This study generated a performance space diagram to evaluate the different asphalt mixtures. In Figure 3, the different areas represent the different performances. The mixture with the best performance has points located in the excellent balance area. If the points are located in the good balance area, the mixture could be selected based on the environment and requirements. Those with points in the worst balance area should be avoided.

#### 3.1.1. Performance Space Analysis for Asphalt Content Optimization

**Figure 4:**
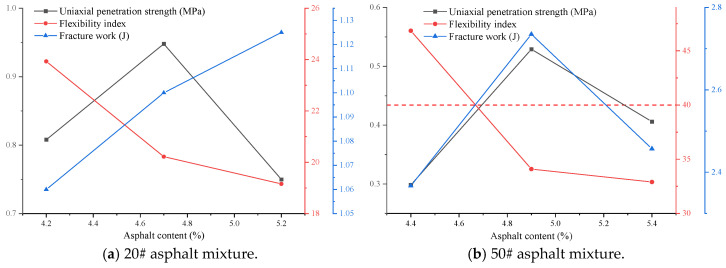
AC-16 mixture 3-y-axes performance space diagrams.

The 3-y-axes performance space diagrams for AC-16 (20# and 50#) are shown in Figure 4.

These results indicate that the low-temperature fracture work of the 20# AC-16 asphalt mixture remained at a good level at all asphalt contents, while its uniaxial penetration strength and flexibility index were both above the “good” level, which is consistent with the trend reported by Zhang et al. [31]. For the 50# AC-16 asphalt mixture, the uniaxial penetration strength did not meet the standard requirements at any asphalt content, whereas the flexibility index reached an excellent level at a 4.4% asphalt content and remained good at the other content levels. In addition, the low-temperature fracture work of the 50# AC-16 asphalt mixture was consistently excellent across all asphalt contents, which is consistent with the findings reported by Zhang et al. [32]: low-grade hard asphalt HMAC exhibits the same low-temperature cracking resistance as conventional HMAC, requiring a higher asphalt film thickness and VMA.

**Figure 5:**
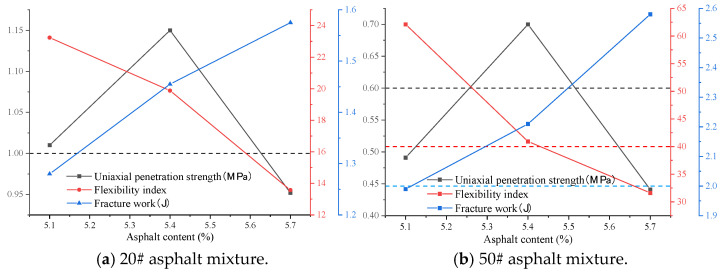
BBME-13 mixture 3-y-axes performance space diagrams.

The 3-y-axes performance space diagrams for BBME-13 (20# and 50#) are shown in Figure 5.

The results show that the uniaxial penetration strength and flexibility index all satisfied the standard. The uniaxial penetration strength of the 5.1% and 5.4% 20# BBME-13 mixtures was excellent while that of the 5.7% mixture was good. The flexibility indexes at all contents were good. The fracture work of the 20# and 50# BBME-13 mixtures at all asphalt contents was good. The uniaxial penetration strength of 50# BBME-13 at 5.4% was good, while the others did not pass the standard. The flexibility index of the 5.1% and 5.4% mixtures was excellent, while the 5.7% mixture showed a good flexibility index.

**Figure 6:**
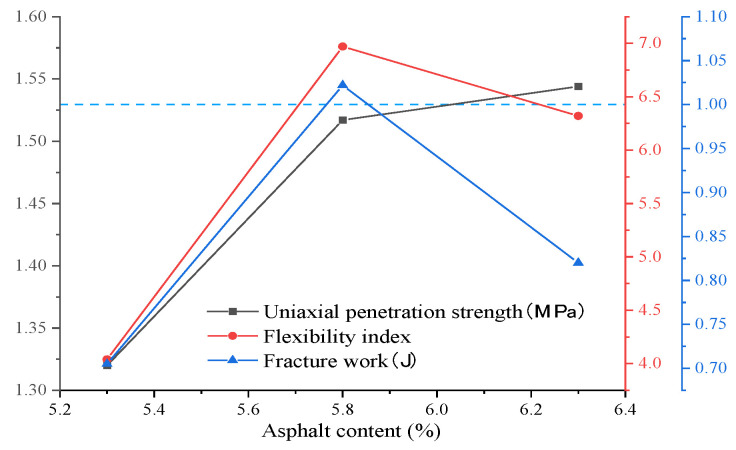
20# EME-20 3-y-axes performance space diagram.

The 3-y-axes performance space diagram for the 20# EME-20 mixture is shown in Figure 6.

The uniaxial penetration strength of EME-20 was excellent but its flexibility index was poor. The fracture work of the 5.8% mixture was good but the others were poor.

#### 3.1.2. Analysis of Distress Interactions

**Figure 7:**
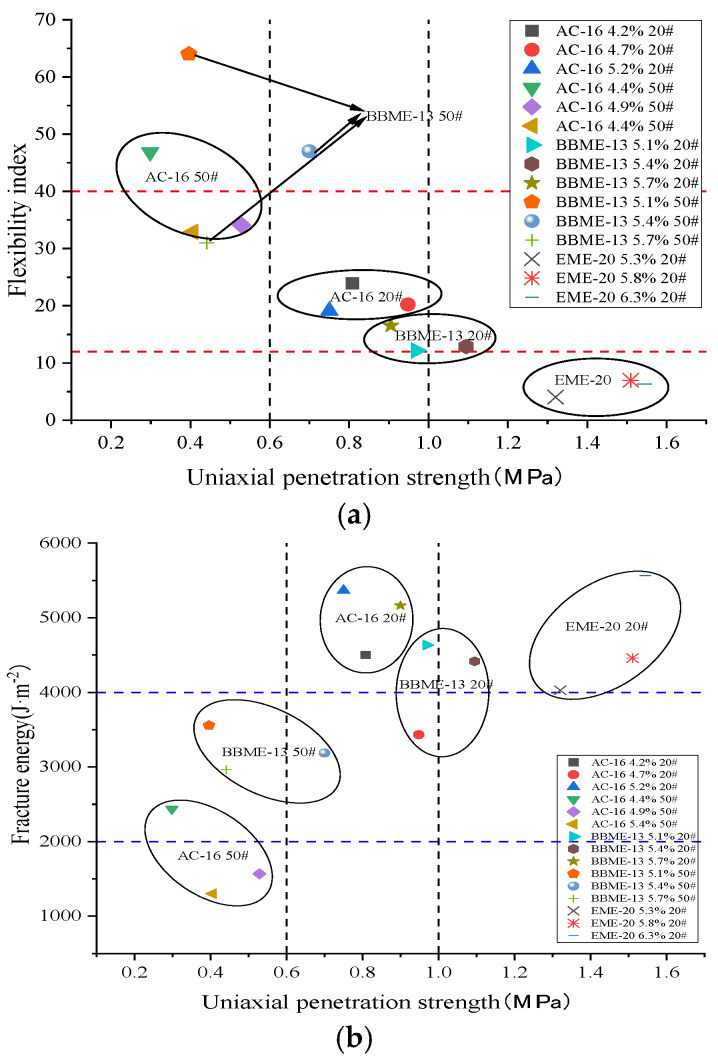

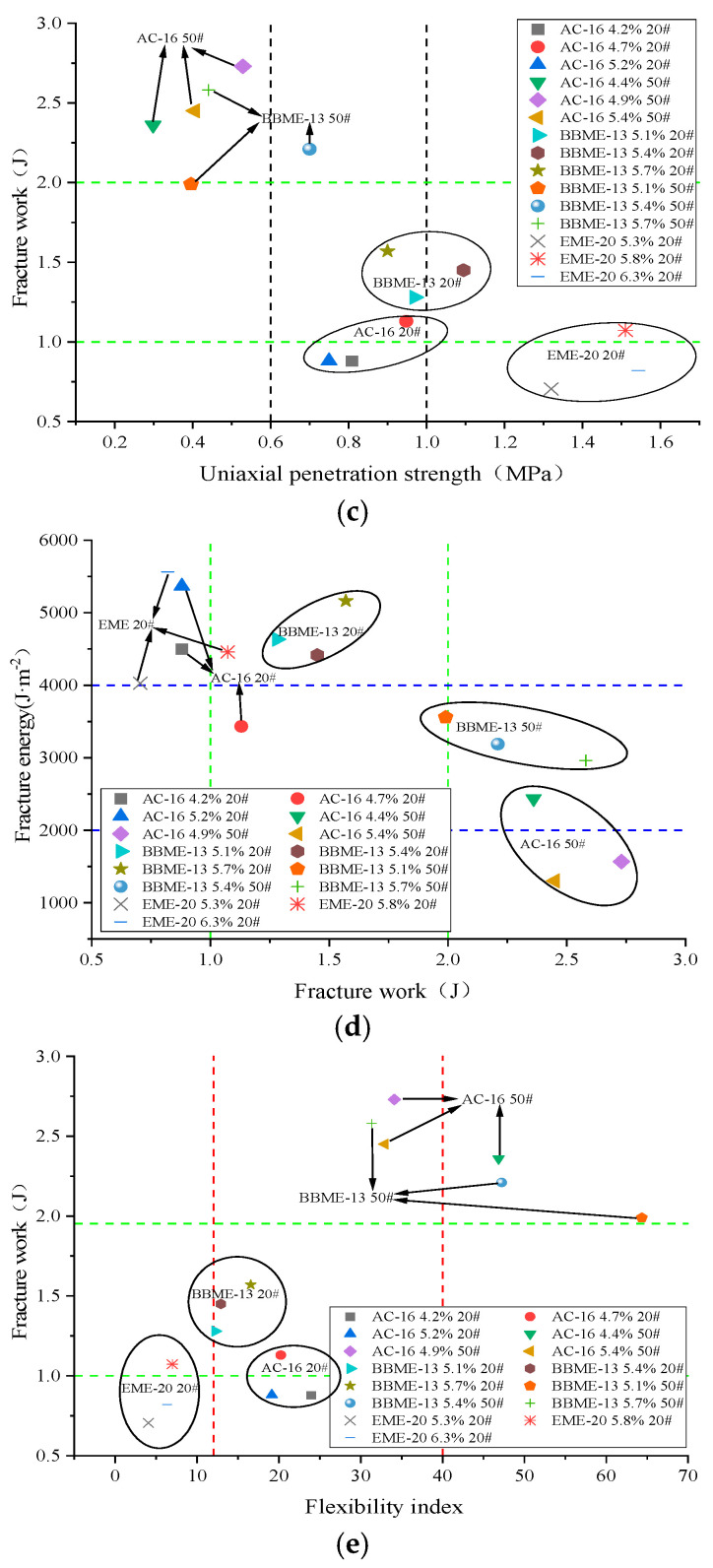
Performance space diagrams. (**a**) Uniaxial penetration strength–flexibility index performance space diagram. (**b**) Uniaxial penetration strength–fracture energy performance space diagram. (**c**) Uniaxial penetration strength–fracture work performance space diagram. (**d**) Fracture energy–fracture work performance space diagram. (**e**) Flexibility index–fracture work performance space diagram.

The results of the balanced analysis of the performance space diagrams are shown in Figure 7.

The uniaxial penetration test results in Figure 7a show that none of the mixtures exhibited excellent strength and flexibility simultaneously. The 20# AC-16 and 20# BBME-13 mixtures demonstrated good uniaxial penetration strength and flexibility index, indicating satisfactory high-temperature shear resistance and medium-temperature crack propagation resistance. In contrast, the 50# AC-16 (4.4%) and 50# BBME-13 (5.1%) mixtures showed excellent flexibility indexes but failed to meet the strength criterion. The EME-20 mixture exhibited the highest uniaxial penetration strength; however, its flexibility index was the lowest among all tested mixtures. Notably, although all 50# mixtures outperformed the 20# mixtures regarding flexibility, only the 50# BBME-13 (5.4%) mixture achieved an adequate uniaxial penetration strength, while the others did not meet the requirement. By comparison, all 20# mixtures possessed good to excellent uniaxial penetration strength, with most also exhibiting satisfactory flexibility.

In Figure 7b, the 50# AC-16 mixture shows the worst uniaxial penetration strength and fracture energy while 50# BBME-13 shows good fracture energy but the worst uniaxial penetration strength. When the asphalt grade was decreased form 50# to 20#, the uniaxial penetration strength and fracture energy improved. The 20# EME-20 and 20# BBME-13 5.4% mixtures had balanced uniaxial penetration strength and fracture energy.

In Figure 7c, although the 50# mixtures show the best fracture work, their uniaxial penetration strength did not pass the criterion. The 20# EME-20 mixtures had the best uniaxial penetration strength but the worst fracture work.

In Figure 7d, when the asphalt grade decreased from 50# to 20#, the low-temperature fracture work was reduced, while the middle-temperature fracture energy improved. Most mixtures did not have balanced fracture energy and fracture work. The 20# mixture had the best middle-temperature fracture energy, but its low-temperature fracture work was good and poor. The 50# mixture had the best low-temperature fracture work, but its middle-temperature fracture energy was good and poor. The 20# BBME-13 mixture had the best fracture energy and good fracture work while the 50# BBME-13 mixtures had the best fracture work and good fracture energy.

In Figure 7e, the 50# group exhibited the best balance between fracture work and flexibility. Specifically, the 50# AC-16 (4.4%) and 50# BBME-13 (5.4%) mixtures demonstrated the best combined performance for both metrics. In contrast, the 20# mixtures showed a poorer balance between fracture work and flexibility, with the 20# EME-20 mixture exhibiting the lowest overall performance in this regard.

In summary, the 20# mixture had the best balance between uniaxial penetration strength and flexibility index; uniaxial penetration strength and fracture energy; and uniaxial penetration strength and fracture work. The 50# mixture had the best balance between fracture work and flexibility index.

### 3.2. Comprehensive Balanced Evaluation and Optimal Mixture Selection

**Figure 8:**
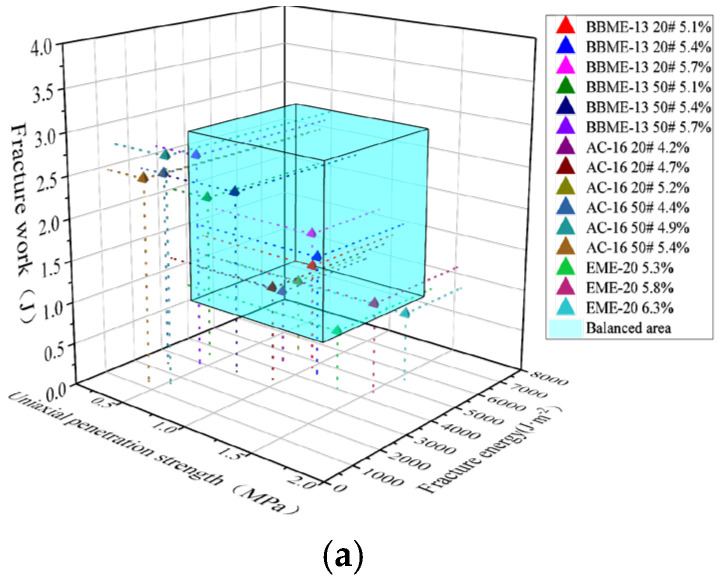

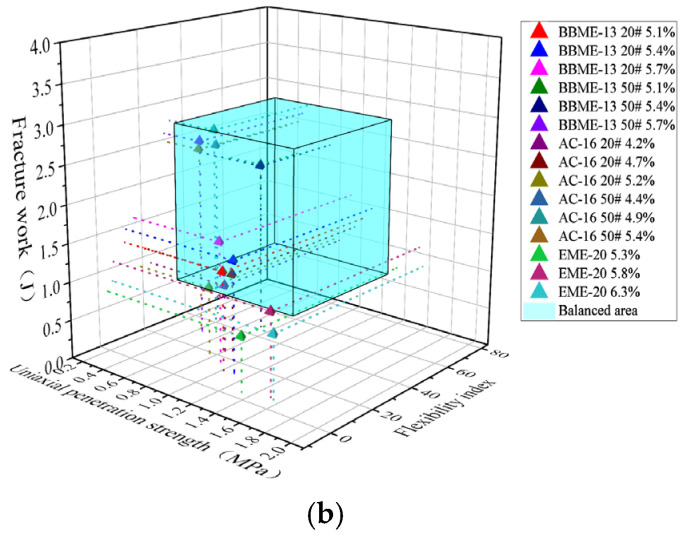
3D performance space diagrams. (**a**) Uniaxial penetration strength–fracture energy–fracture work. (**b**) Uniaxial penetration strength–fracture energy–fracture work.

The 3D performance space diagrams obtained from the multi-performance balanced analysis are shown in Figure 8.

The good performance area was determined based on the results. The 20# BBME-13 (5.1%, 5.4%, and 5.7%), 50# BBME-13 5.4%, and 20# AC-16 4.7% mixtures had well-balanced performances. Therefore, these mixtures were used to design upper layer mixtures.

The final asphalt contents of the 20# AC-16, 50# AC-16, 20# BBME-13, 50# BBME-13, and 20# EME-20 mixtures were 4.7%, 4.9%, 5.6%, 5.4% and 5.9%. A semi-circle bending fatigue test under stress control was conducted; the results are shown in Table 10.

**Figure 9:**
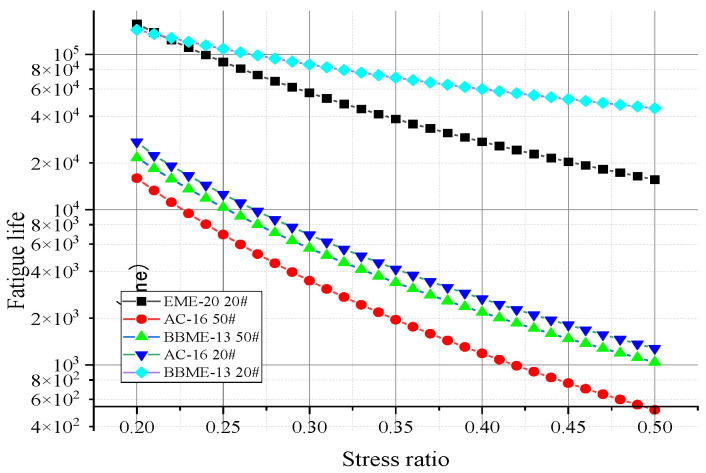
Fatigue curves.

The BBME-13 and EME-20 mixtures had obviously higher fatigue lives than the AC-16 mixtures, and the 20# mixtures had obviously higher fatigue lives than the 50# mixtures. The fatigue life results are shown in Table 3, with the fitted curves shown in Figure 9.

The BBME-13 and EME-20 mixtures had higher fatigue lives and lower sensitivities to stress. The 20# BBME-13 and 20# EME-20 mixtures had the highest fatigue lives. For the BBME-13 and AC-16 gradations, reducing the asphalt grade significantly improved the fatigue life and decreased the sensitivity to stress. Under a low stress ratio, 20# EME-20 had the best fatigue performance, while under high stress, 20# BBME-13 had the best fatigue performance. Regarding the BBME-13 mixtures, the *K* value of 20# BBME-13 increased by 143.7% compared with 50# BBME-13, while the *n* value decreased by 61.6%. Regarding the AC-16 mixtures, the K value of 20# AC-16 increased by 1033.0% compared with 50# AC-16, while the *n* value decreased by 13.86%. Compared with 20# AC-16, the *K* value of 20# BBME-13 increased by 241.1% and the *n* value increased by 60.6%. Compared with 50# AC-16, the *K* value of 50# BBME-13 increased by 151.1%, while the *n* value decreased by 11.7%. In addition, the *K* value of 20# EME-20 increased by 59.2% compared with 20# BBME-13, and the *n* value increased by 98.4%. Compared with the literature [33,34], the 20# BBME-13 asphalt mixture had a denser and finer aggregate structure, a more uniform gradation distribution, and a clear load path, thus exhibiting optimal fatigue performance. Compared with the AC-16 mixture, BBME-13 had more fine aggregate and higher asphalt content, forming a mortar with a higher strength. In addition, BBME-13 had a lower air void content, resulting in a denser coarse aggregate structure. Therefore, the BBME-13 mixture had better fatigue performance than the AC-16 mixture.

## 4. Conclusions

This paper presents a performance space diagram (PSD)-based balanced mix design approach, which is a simple yet effective method for simultaneously evaluating the high-, intermediate-, and low-temperature performances of high-modulus asphalt mixtures regarding mixture design, optimization, and performance verification. The proposed framework incorporates uniaxial penetration strength, semi-circular bending flexibility index, and semi-circular bending fracture energy as key performance indicators, in a manner analogous to the control of volumetric parameters in traditional mix design methods. In addition, other performance tests, such as fatigue tests, can be incorporated into this framework to address other pavement distresses.

The PSD approach involves mapping multiple performance indicators onto coordinate axes, enabling a direct visualization of the balanced performance region of different mixtures in a multi-dimensional performance space. The PSD plots provide valuable insights into the key variables affecting overall mixture performance. In this study, variables such as gradation types from China and France (AC-16, BBME-13, and EME-20), asphalt grades (20# and 50# hard asphalt), asphalt content (determined based on 4% air voids or the abundance coefficient K), and aggregate structural characteristics (dense-graded versus skeleton-dense structures) were considered.

The study can be summarized as follows:

(1) BMD was applied to AC-16, BBME-13 and EME-20 mixtures using uniaxial penetration strength tests, semi-circle bending-flexibility index, semi-circle bending-fracture energy and semi-circle bending fatigue tests, and the corresponding evaluation indexes (uniaxial penetration strength, flexibility index, fracture energy, and fracture work) were used to evaluate the high-, intermediate-, and low-temperature mechanical properties of the asphalt mixtures.
(2) Based on the performance space diagram, the 20# mixture had the best balance between uniaxial penetration strength and flexibility index; uniaxial penetration strength and fracture energy; and uniaxial penetration strength and fracture work. The 50# mixture had the best balance between fracture work and flexibility index, while the 20# BBME-13 (5.1%, 5.4%, and 5.7%), 50# BBME-13 5.4%, and 20# AC-16 4.7% mixtures had well-balanced performances. Thus, these high-modulus asphalt mixtures prepared using low-grade hard asphalt exhibited comparable pavement performance to conventional high-modulus asphalt mixtures while requiring a greater asphalt film thickness.
(3) The BBME-13 and EME-20 mixtures had higher fatigue lives and lower sensitivities to stress than the AC-16 mixture. In addition, the 20# BBME-13 and 20# EME-20 mixtures had the best fatigue performance. For the BBME-13 and AC-16 mixtures, reducing the asphalt grade significantly increased fatigue life and decreased sensitivity to stress. Under low stress, the 20# EME-20 mixture had the best fatigue performance, while under high stress, the 20# BBME-13 mixture had the best fatigue performance.

## Figures and Tables

**Table 1:** Technology properties of hard asphalt.

Asphalt Type	Penetration (0.1 mm)			Penetration Index *PI*	Softening Point (°C)	Ductility (cm)		
	15 °C	25 °C	30 °C			5 °C	15 °C	25 °C
20#	7.2	16.8	25.7	0.56	70.0	0.0	4.6	12.2
50#	21.1	55.1	87.3	−0.21	51.9	12.3	>150	>150
Asphalt technological indexes after RTFOT								
Asphalt type	Mass change fraction (%)		Penetration (25 °C, 0.1 mm)		Residue penetration ratio (%)		Ductility (15 °C, cm)	
20#	0.054		15.2		89.6		0.0	
50#	−0.810		31.7		57.2		9.4	

**Table 2:** Aggregate properties.

Parameter	Unit	10~20 mm	5~10 mm	3~5 mm	0~3 mm	Mineral Powder
Bulk volume relative density	-	2.713	2.570	2.433	2.616	2.730
Apparent relative density	-	2.807	2.666	2.676	2.617	2.730
Crushing value	%	21.7	/	/	/	/
Los Angeles abrasion loss	%	18.8	18.8	/	/	/
Water absorption rate	%	0.774	1.051	0.641	0.84	/
Elongated and flaky particle content	%	10.8	6.7	/	/	/
Soundness	%	/	/	/	6.4	/
Methylene blue value	g/kg	/	/	/	4.6	/
Sand equivalent	%	/	/	/	64	/
Proportion		30	17	8	40	5

**Table 3:** Gradation composition of asphalt mixture (unit: %).

Mixture Type	Passing Rate (%)									
	16	13.2	9.5	4.75	2.36	1.18	0.6	0.3	0.15	0.075
AC-16	95	84	70	45.2	34	24.5	17.5	12.5	9.5	6

**Table 4:** Volumetric parameters of 20# AC-16 mixture.

Volumetric Parameter	Asphalt Mixture		
	4.0% Asphalt Content	4.5% Asphalt Content	5.0% Asphalt Content
Bulk volume relative density	2.392	2.401	2.411
Theoretical maximum density	2.622	2.511	2.463
Voids in the mineral aggregate (%)	12.56	12.69	12.78
Voids filled with asphalt (%)	30.16	65.47	83.48
Air voids (%)	8.77	4.38	2.11

**Table 5:** Volumetric parameters of 50# AC-16 mixture.

Volumetric Parameter	Asphalt Mixture		
	4.0% Asphalt Content	4.5% Asphalt Content	5.0% Asphalt Content
Bulk volume relative density	2.280	2.284	2.307
Theoretical maximum density	2.469	2.421	2.392
Voids in the mineral aggregate (%)	16.65	16.94	16.55
Voids filled with asphalt (%)	54.11	66.60	78.52
Air voids (%)	7.64	5.67	3.55

**Table 6:** Gradation composition of each asphalt mixture (unit: %).

Mixture Type	Sieve Size (mm)										
	20	16	13.2	9.5	4.75	2.36	1.18	0.6	0.3	0.15	0.075
BBME-13	/	95	84	70	48	34	24.5	17.5	7	5	6
EME-20	95	85	71	62	49	35.5	26.2	16	11	8.5	6.9

**Table 7:** Abundance coefficients for BBME-13 and EME-20.

Gradation	Abundance Coefficient Indexes							
	*G* (%)	*S* (%)	*S* (%)	*F* (%)	*G_se_*	*α*	*∑*	*K*
BBME-13	57.199	48.839	3.152	5.208	2.686	0.986	8.67	3.3
EME-20	44.1	46.01	3.75	5.76	2.690	0.985	9.40	3.4

**Table 8:** Volumetric parameters.

Volumetric Parameter	20# BBME-13			50# BBME-13			EME-20		
	5.1%	5.4%	5.7%	5.1%	5.4%	5.7%	5.3%	5.8%	6.3
Bulk volume relative density	2.330	2.410	2.401	2.323	2.346	2.353	2.401	2.409	2.411
Theoretical maximum density	2.473	2.469	2.466	2.465	2.465	2.464	2.49	2.49	2.49
Voids in the mineral aggregate (%)	16.07	13.19	13.51	16.32	15.49	15.24	13.05	13.46	14.17
Voids filled with asphalt (%)	64.10	81.88	80.49	64.70	68.84	69.09	75.69	77.62	80.44
Air voids (%)	5.78	2.39	2.64	5.76	4.83	4.50	3.17	3.01	2.77

**Table 9:** Performance tests.

Test Method	Test Conditions	Specimen Size	Test Index	Expression	Explanation
Uniaxial penetration test	Temperature: 60 °C; Loading rate: 50 mm/min	Cylinder: 100 mm diameter and 100 mm height	Uniaxial penetration strength $\sigma_{p}$	$\sigma_{p}=0.34*\frac{P}{A}$	P is the peak load; A is the cross-sectional area of the pressure head
Semi-circle bending test (flexibility index)	Temperature: 25 °C; Loading rate: 50 mm/min	Semi-circle: 100 mm diameter, 50 mm width, and 15 mm cut	Peak load (P) and flexibility index (FI)	$FI=\frac{G_{f}}{\left\lceil m\right\rceil }*10^{6}$ Peak load	$G_{f}$ is the fractureenergy; ⌈m⌉ is the slope after the peak load
Semi-circle bending test (fracture energy)	Temperature: −10 °C; Loading rate: 1 mm/min	Semi-circle: 100 mm diameter, 25 mm width, and 15 mm cut	Fracture work $(W_{f}$)	$W_{f}=\int_{0}^{u_{0}}P(u)du$	$u_{0}$ is the displacement under a 0.5 kN load after the peak load; P(u) is the function of displacement and load
Semi-circle bending test (fatigue)	Temperature: 15 °C; Loading frequency: 15 Hz; Stress ratios: 0.2, 0.3, 0.4, and 0.5	Semi-circle: 100 mm diameter, 40 mm width, and 15 mm cut	Fitting parameters: K and *n*	$N_{f}=K{\left(\frac{1}{\sigma_{0}}\right)}^{n}$	$N_{f}$ is the number of repeated loadings before the specimen broke; $\sigma_{0}$ is the initial stress; *K* and *n* are the fitting parameters

**Table 10:** Fatigue test results.

Mixture Type	Stress Ratio	Initial Load (kN)	Stress (MPa)	Fatigue (Time)	*K*	*n*	R^2^
20# BBME-13	0.2	1.10	0.88	136,341	122,552.47	1.27	0.90
	0.3	1.66	1.33	104,795			
	0.4	2.21	1.77	74,501			
	0.5	2.77	2.21	12,415			
50# BBME-13	0.2	0.93	0.74	21,400	7968.99	3.31	0.99
	0.3	1.39	1.11	5417			
	0.4	1.85	1.48	2007			
	0.5	2.32	1.85	1628			
20# AC-16	0.2	1.36	1.088	27,206	35,913.83	3.23	0.99
	0.3	2.04	1.632	8534			
	0.4	2.72	2.176	1523			
	0.5	3.40	2.72	897			
50# AC-16	0.2	0.82	0.65	15,170	3169.67	3.75	0.99
	0.3	1.23	0.98	3282			
	0.4	1.64	1.31	965			
	0.5	2.05	1.64	885			
20# EME-20	0.2	1.36	1.09	153,224	195,163.4	2.52	0.97
	0.3	2.04	1.63	75,527			
	0.4	2.73	2.18	14,235			
	0.5	3.41	2.72	1861			

## Data Availability

The original contributions presented in this study are included in the article. Further inquiries can be directed to the corresponding authors.

## References

[1] Lee H.J., Lee J.H., Park H.M. (2006). Performance evaluation of high modulus asphalt mixtures for long life asphalt pavements. Constr. Build. Mater..

[2] Rys D., Judycki J., Pszczola M., Jaczewski M., Mejlun L. (2017). Comparison of low-temperature cracks intensity on pavements with high modulus asphalt concrete and conventional asphalt concrete bases. Constr. Build. Mater..

[3] Haritonovs V., Tihonovs J., Smirnovs J. (2016). High modulus asphalt concrete with dolomite aggregates. Transp. Res. Arena.

[4] Ullah A., Wen H., Ullah Z., Ali B., Khan D. (2024). Evaluation of high modulus asphalts in China, France, and USA for durable road infrastructure, a theoretical approach. Constr. Build. Mater..

[5] Xia C., Cong B., Lv S., Wang D., Liu B., Zhao T., Jiang X. (2025). Characterization of fatigue damage in high-modulus asphalt mixtures at different aging degrees. Constr. Build. Mater..

[6] Wang Z., Shu C., Han B., Fu Y., Zhou L. (2020). Research progress of high modulus asphalt concrete. J. Chang’an Univ. (Nat. Sci. Ed.).

[7] Randy W., Rodezno C., Fabricio L., Fan Y. (2018). Development of a Framework for Balanced Mix Design.

[8] Li N., Hao P., Yao Y., Zhang C. (2023). The implementation of balanced mix design in asphalt materials: A review. Constr. Build. Mater..

[9] Ziari H., Hajiloo M. (2023). The effect of mix design method on performance of asphalt mixtures containing reclaimed asphalt pavement and recycling agents: Superpave versus balanced mix design. Case Stud. Constr. Mater..

[10] (2020). Standard Practice for Balanced Design of Asphalt Mixtures.

[11] Diefenderfer S., Bowers B. (2019). Initial Approach to Performance (Balanced) Mix Design: The Virginia Experience. Transp. Res. Rec..

[12] Nsengiyumva G., Kim Y.-R., Hu J. (2020). Feasibility and Implementation of Balanced Mix Design in Nebraska. https://digitalcommons.unl.edu/ndor/258/.

[13] Cooper S.B., Mohammad L.N., Kabir S., King W. (2014). Balanced Asphalt Mixture Design through Specification Modification: Louisiana’s Experience. Transp. Res. Rec..

[14] Zhou F., Scullion T., Walubita L., Wilson B. (2014). Implementation of a performance-based mix design system in Texas. Transp. Res. Circ..

[15] Zhang Z., Luo Y., Zhang K. (2017). Review on hamburg wheel-track device evaluation of asphalt mixture. Mater. Rev..

[16] Liu L., Zhang X. (2018). The correlation between the high temperature indicators of theasphalt and shearing properties of the mixture. J. Transp. Sci. Eng..

[17] Wu B., Liu L., Sun L. (2019). Influence of different parameters on shear performance of asphalt mixture. J. Highw. Transp. Res. Dev..

[18] Jalkh R., El-Rassy H., Chehab G., Abiad M. (2018). Assessment of the Physico-Chemical Properties of Waste Cooking Oil and Spent Coffee Grounds Oil for Potential Use as Asphalt Binder Rejuvenators. Waste Biomass Valorization.

[19] Buttlar W., Hill B., Wang H., Mogawer W. (2017). Performance space diagram for the evaluation of high- and low-temperature asphalt mixture performance. Road Mater. Pavement Des..

[20] Rath P., Love J.E., Buttlar W.G., Reis H. (2019). Performance Analysis of Asphalt Mixtures Modified with Ground Tire Rubber Modifiers and Recycled Materials. Sustainability.

[21] (2025). Standard Test Methods of Bitumen and Bituminous Mixturesfor Highway Engineering. The People’s Republic of China.

[22] (2025). Test Methods of Aggregates for Highway Engineering. The People’s Republic of China.

[23] (2004). Technical Specification for Construction of Highway Asphalt Pavements. The People’s Republic of China.

[24] Delorme J.L., Chantal D.L.R., Wendling L. (2007). LPC Bituminous Mixtures Design Guide.

[25] Chen H., Fan T.J., Fan F.F. (2012). Influence of Specimen Thickness on ShearPerformance of Asphalt Mixture in Uniaxial Penetration Test. J. Chongqing Jiaotong Univ. (Nat. Sci.).

[26] Al-Qadi I.L., Ozer H., Lambros J., El Khatib A., Singhvi P., Khan T., Rivera-Perez J., Doll B. (2015). Testing Protocols to Ensure Performance of High Asphalt Binder Replacement Mixes Using RAP and RAS.

[27] El Khatib A.K. (2016). Development of a Performance-Based Test Method for Quantification of Cracking Potential in Asphalt Pavement Materials.

[28] Chen X., Li W., Li H. (2009). Evaluation of fracture properties of epoxy asphalt mixtures by SCB test. J. Southeast Univ. (Engl. Ed.).

[29] Jiang J., Ni F., Dong Q., Wu F., Dai Y. (2018). Research on the fatigue equation of asphalt mixtures based on actual stress ratio using semi-circular bending test. Constr. Build. Mater..

[30] Jiang J., Ni F., Dong Q., Zhao Y., Xu K. (2018). Fatigue damage model of stone matrix asphalt with polymer modified binder based on tensile strain evolution and residual strength degradation using digital image correlation methods. Measurement.

[31] Zhang Z. (2015). Experimental study on performance of 20# hard asphalt mixture. J. Highw. Transp. Res. Dev..

[32] Zhang Y., Cheng H., Sun L., Liu L., Hu Y. (2021). Determination of volumetric criteria for designing hard asphalt mixture. Constr. Build. Mater..

[33] Xu X.Z. (2020). Study on the Structure and Material Design of Full-Depth High Modulus Asphalt Pavement. Master’s Thesis.

[34] Ma J. (2011). Research on Characters of High Modulus Asphalt Concrete Pavementin Heavy Traffic Condition. Ph.D. Thesis.

